# High-Intensity Interval Training Attenuates Insulin Resistance Induced by Sleep Deprivation in Healthy Males

**DOI:** 10.3389/fphys.2017.00992

**Published:** 2017-12-07

**Authors:** Jorge F. T. de Souza, Murilo Dáttilo, Marco T. de Mello, Sergio Tufik, Hanna K. M. Antunes

**Affiliations:** ^1^Departamento de Biociências, Universidade Federal de São Paulo, São Paulo, Brazil; ^2^Centro de Estudos em Psicobiologia e Exercício, São Paulo, Brazil; ^3^Departamento de Psicobiologia, Universidade Federal de São Paulo, São Paulo, Brazil; ^4^Departamento de Esportes, Faculdade de Educação Física, Fisioterapia e Terapia Ocupacional, Universidade Federal de Minas Gerais, Belo Horizonte, Brazil

**Keywords:** high-intensity interval training, sleep deprivation, insulin resistance, glucose metabolism, physical exercise

## Abstract

**Introduction:** Sleep deprivation can impair several physiological systems and recently, new evidence has pointed to the relationship between a lack of sleep and carbohydrate metabolism, consequently resulting in insulin resistance. To minimize this effect, High-Intensity Interval Training (HIIT) is emerging as a potential strategy.

**Objective:** The aim of this study was to investigate the effects of HIIT on insulin resistance induced by sleep deprivation.

**Method:** Eleven healthy male volunteers were recruited, aged 18–35 years, who declared taking 7–8 h sleep per night. All volunteers were submitted to four different conditions: a single night of regular sleep (RS condition), 24 h of total sleep deprivation (*SD* condition), HIIT training followed by regular sleep (HIIT+RS condition), and HIIT training followed by 24 h of total sleep deprivation (HIIT+*SD* condition). They performed six training sessions over 2 weeks and each session consisted of 8–12 × 60 s intervals at 100% of peak power output. In each experimental condition, tests for glucose, insulin, cortisol, free fatty acids, and insulin sensitivity, measured by oral glucose tolerance test (OGTT), were performed.

**Results:** Sleep deprivation increased glycaemia and insulin levels, as well as the area under the curve. Furthermore, an increase in free fatty acids concentrations and basal metabolism was observed. There were no differences in the concentrations of cortisol. However, HIIT before 24 h of sleep deprivation attenuated the increase of glucose, insulin, and free fatty acids.

**Conclusion:** Twenty-four hours of sleep deprivation resulted in acute insulin resistance. However, HIIT is an effective strategy to minimize the deleterious effects promoted by this condition.

## Introduction

The decrease in total sleep time, the increase in sleep complaints and consequently, sleep deprivation are more frequent nowadays (Santos-Silva et al., 2010). Several studies show that sleep is able to influence the responses of β pancreatic cells and the sensitivity of tissues to insulin (Ip and Mokhlesi, 2007; Knutson et al., 2007). On the other hand, lack of sleep can negatively influence glucose homeostasis resulting in insulin resistance (González-Ortiz et al., 2000; Tasali et al., 2008; Nedeltcheva et al., 2009; Buxton et al., 2010; Donga et al., 2010; Klingenberg et al., 2013).

Among the strategies that can minimize the negative impact of insulin resistance, regular physical exercise emerges as a non-pharmacological strategy offering health benefits for the general population (Garber et al., 2011) and acts directly on the regulation of glucose metabolism from acute and chronic effects, activating the insulin signaling pathway and the independent pathway of insulin action (Lima et al., 2009; Pauli et al., 2009). In this scenario, High-Intensity Interval Training (HIIT) appears to be a time-efficient strategy capable of providing the same benefits as traditional moderate-intensity continuous exercise but with less expenditure of practice time. The effects are related to increased insulin sensitivity and improved glycaemic control (Babraj et al., 2009; Richards et al., 2010).

Modern society is becoming increasingly fast-paced, and the time devoted to sleep and physical exercise is correspondingly limited, thus HIIT could be beneficial in reversing or minimizing the change in glucose metabolism caused by sleep deprivation. Therefore, this study aimed to investigate the effects of HIIT on insulin resistance as a result of sleep deprivation. The initial hypothesis was that people deprived of sleep for 24 h following a 2-week period of HIIT, would suffer less from the deleterious effects of sleep debt.

## Materials and methods

Eleven healthy and physically active young men were recruited by advertisements in flyers, radio, newspaper, and social network website. The subjects were selected for the study based on the following inclusion criteria: (a) male gender; (b) aged between 18 and 30 years; (c) physically active (aerobic modalities for least 2 years, 5x/week); (d) habitual sleep for 7–8 h/night; (e) regular eating habits; (f) no cardiovascular disease, diabetes mellitus, or impaired glucose tolerance; (g) no physical injuries or disabilities; (h) non-smoker; (i) consuming no more than 2 doses/day of alcohol and no more than 4 doses on a single occasion; (j) no chronic use of anti-inflammatory and antilipidemic medications; (k) no sleep disorders; and (l) no alterations on the electrocardiogram at rest and during exercise.

Before participating, all volunteers were informed of the procedures, discomfort, and risks involved in the evaluation process. This study was carried out in accordance with the recommendations of the ethics committee of institution with written informed consent from all subjects. All subjects gave written informed consent in accordance with the Declaration of Helsinki. The Committee of Ethics in Research of the Universidade Federal de São Paulo/Hospital São Paulo approved the study (#522.163) and the study was registered at Clinical Trials (NCT02125656).

### Experimental design

During their first visit to the laboratory and before initiating the protocol, all volunteers underwent a clinical evaluation that included a resting electrocardiogram and an ergometric test, all conducted by a specialist doctor, including a thorough explanation of study procedures and the completion of the informed consent form. After these evaluations, only those subjects considered healthy participated in the study. During their second visit, volunteers received a full overnight, attended polysomnography using a computerized system (EMBLA®S7000, Embla Systems, Inc., Broomfield. CO, USA). Following the night that the PSG was performed, four sleep questionnaires (Epworth Sleepiness Scale, Pittsburgh Sleep Quality Index, UNIFESP Sleep Questionnaire and Mini-Sleep Questionnaire) were applied to complement the evaluation of the sleep profile. These questionnaires were chosen to complement the information obtained by polysomnography examination, reporting excessive daytime sleepiness, subjective perception of sleep quality, sleep habits and complaint events. The instruments are validated for the Brazilian population. In addition, each volunteer provided blood samples and an OGTT was performed. Each volunteer was instructed in the use of a Motionlogger Actigraph Watch® (Ambulatory Monitoring Inc., Ardsley, NY, USA), to be worn for 7 days preceding each experimental condition, to evaluate the sleep-wake cycle and registration of motor activity from the movements member.

After completing all evaluations, the volunteers were considered ready to start the experiment.

### Experimental protocol

All volunteers were submitted to four different conditions: Regular Sleep (RS), Sleep Deprivation (*SD*), HIIT+Regular Sleep (HIIT+RS), and HIIT+Sleep Deprivation (HIIT+*SD*).

In RS condition, subjects had a regular night's sleep for 8 h. After waking-up, they were submitted to blood analyses and OGTT.

In *SD* condition, subjects were submitted to 24 consecutive hours of sleep deprivation. Throughout this period, the volunteers had access to television, music and video games, and fasted during the night. In the morning, at same time as other experiments, blood samples were taken for analysis and an OGTT was carried out.

In HIIT+RS condition, subjects trained for 2 weeks, and after the last day of training, had a regular night's sleep. After waking-up, full blood analyses and an OGTT were carried out.

**Figure 1:**
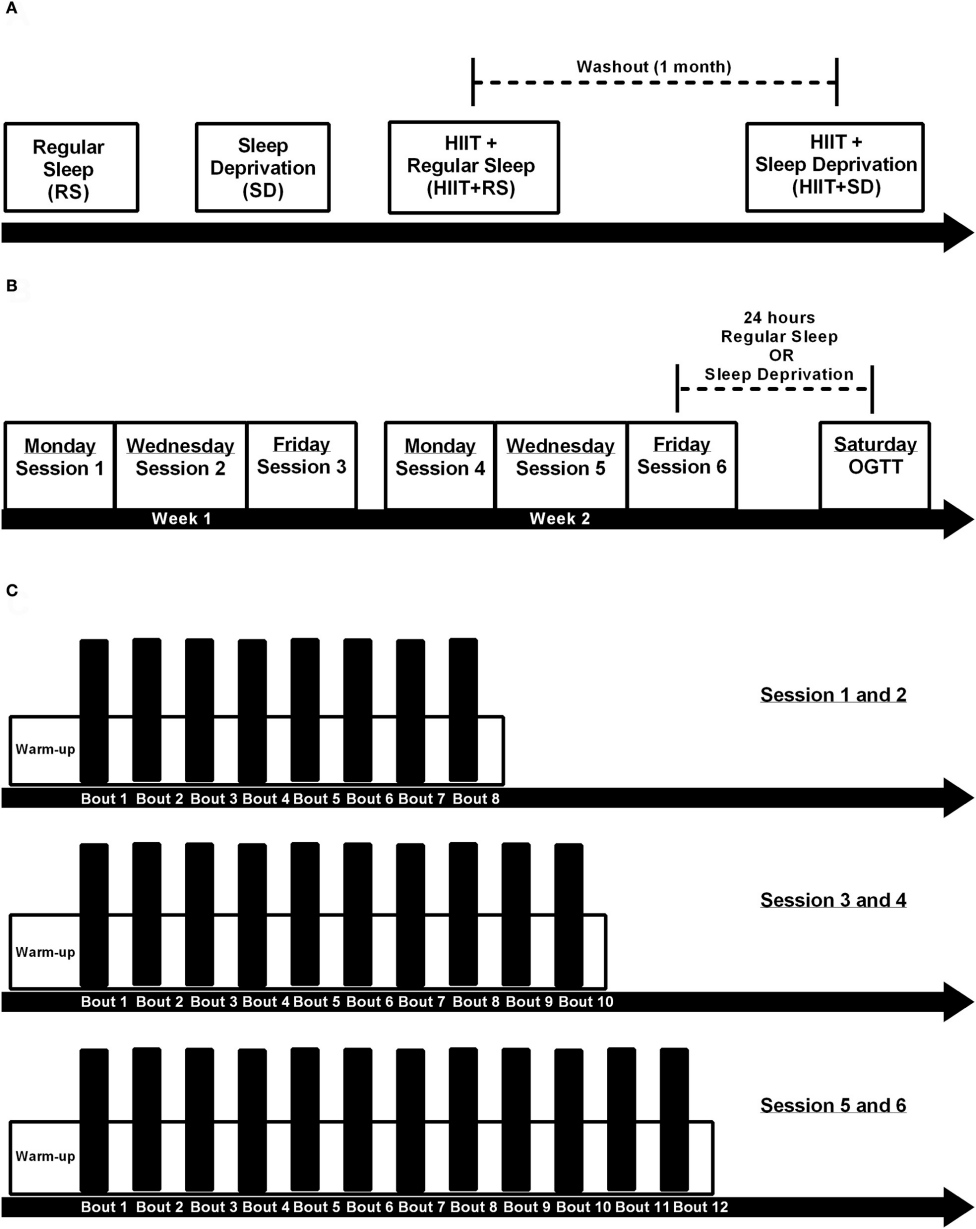
Experimental design. **(A)** Order of experimental conditions, where first the volunteers were submitted a single night of regular sleep (RS condition), second the volunteers were submitted 24 h sleep deprivation (*SD* condition), third the volunteers were submitted 2 weeks of HIIT training and after last session had a night of regular sleep (HIIT+RS condition), and after a month of washout the volunteers repeated the 2 weeks of HIIT training and after last session had a 24 h sleep deprivation (HIIT+*SD* condition); **(B)** Each training session distributed over 2 weeks; **(C)** Design of 1-6 HIIT training sessions.

After a 1-month washout period, all volunteers returned to the labs to start HIIT+*SD* condition. They repeated the training protocol and immediately after training finished, the volunteers were deprived of sleep for 24 consecutive hours and in the morning, full blood analyses, and OGTT were performed (see Figure 1).

### Questionnaires

International Physical Activity Questionnaire Short Form (IPAQ-SF)—used to determine the physical activity level. The questionnaire had been previously translated into Portuguese and validated for use by Brazilian participants (Matsudo et al., 2001). Questions related to the activities undertaken in the previous week. Volunteers answered 9 questions about walking frequency and duration, the frequency of taking moderate or vigorous physical activity, and estimates of sedentary time per 7-day week. The physical activity level was categorized using the recommendations established by the World Health Organization (Guilbert, 2003). Participants were considered physically active if they performed at least 150 min of physical activity per week (including time spent at work, traveling to/from work, household chores, and leisure time). Participants who performed between 10 and 149 min of physical activity were considered moderately active, and those who performed < 10 min per week were classified as insufficiently active.

Epworth Sleepiness Scale—used to assess daytime sleepiness, this scale assesses the probability of dozing off in eight everyday situations by means of a scale ranging from 0 (= would never doze) to 3 (= high chance of dozing). Scores >10 are considered as elevated and clinically significant (Bertolazi et al., 2009).

Pittsburgh Sleep Quality Index—used to assess sleep quality over the previous month. A total of 18 items, each scored from 0 to 3, generate different subscales: subjective sleep quality, sleep onset latency, sleep duration, habitual sleep efficiency, sleep disturbances, use of sleep medication, and daytime dysfunction. The total score is calculated by summing all subscale scores with a total of >5 indicating “bad sleepers,” in comparison to < 5 for “good sleepers.” A total score >10 indicates a several sleep problems or sleep disorders (Bertolazi et al., 2011).

UNIFESP Sleep Questionnaire—used to evaluate sleep habits and sleep complaints (Pires et al., 2007).

Mini-Sleep Questionnaire—a self-reported questionnaire about current sleep quality, which is has ten questions about the frequency of sleep difficulties. Answer options range from 1 (never) to 7 (always). The total score is classified into good sleep (10–24), mild sleep difficulties (25–27), moderate difficulties (28–30), and severe difficulties (>30) (Falavigna et al., 2011).

### Training

The training protocol was based on the work of Little et al. (2010), which consists of four phases: maximal test, pre-training test, 2 weeks of HIIT and post-training tests.

### Maximal test

Incremental exercise was performed until maximum volitional exhaustion, using a cycle ergometer (Excalibur Sport 925900, Lode BV, Groningen, The Netherlands), in a set hyperbolic mode to determine VO_*2peak*_. In this protocol, the initial load was set at 70 W and then increased by 35 W every 2 min. Throughout the test, volunteers were verbally encouraged and required to maintain a minimum rate of 70 RPM. Ventilatory parameters were obtained by measuring respiratory gas exchange with a metabolic system (Quark PFT 4 Ergo, COSMED®, Rome, Italy).

### Pre-training tests

Two days after the maximal test, the volunteers performed two time-trial cycling tests, separated by 48 h. They were instructed to complete the test as quickly as possible without verbal encouragement or physiological feedback. They had visual feedback only about the distance that appeared on the computer monitor. At the end of each test, the result was presented in units of distance, rather than work completed (4 km was displayed to represent 100 kJ, and 30 km was displayed to represent 750 kJ). The ergometer was set to linear mode, so that the resistance increased proportionally to cadence and force.

### Training

Three days after the tests, the volunteers started a training protocol that consisted of six sessions over 2 weeks (Mondays, Wednesdays, and Fridays). Each training session consisted of repeated efforts of high-intensity cycling at a workload corresponding to VO_*2peak*_ (peak power) for 60 s. These sprints were interspersed with an active recovery of 75 s at low intensity (30 W). Each day, before training began, 3 min of warm-up at 30 W was performed. The subjects completed 8 high-intensity intervals during the first two training sessions, 10 intervals during third and fourth sessions, and 12 intervals during the final two sessions. All volunteers completed all training sessions without complications.

### Post-training tests

Seventy-two hours after the final training session, volunteers were submitted to two time-trial tests 48 h apart.

### Oral glucose tolerance test

After an 8 h overnight fast, subjects ingested 75 g of anhydrous glucose dissolved in 300 ml water (GlucUp 75, lemon flavor, Newprov, Pinhais, Brazil). Plasma glucose and plasma insulin levels were tested at 0, 30, 60, 90, and 120 min.

### Food diary

All participants were instructed to maintain their regular eating habits during the study period. To obtain this control, volunteers completed a food diary every day during the training period. The diaries were analyzed quantitatively by calculating energy, macronutrients, and micronutrients, using NutWin-UNIFESP software. The food diary was adopted as a self-control tool for participants' diets, and the data were presented as mean kcal of the training period.

### Biochemical analysis

Blood collection was conducted early in the morning after a fasting period, by surface puncture of the forearm vein, with the volunteers in a seated position. The samples were centrifuged to separate the plasma and serum and were then stored at −80°C until the time of analysis. Insulin and cortisol were determined using immunoassay system (Unicel® DxI800, Access®, Beckman Coulter®), blood glucose was analyzed by the colorimetric/enzymatic assay (Unicel® DxI 800, Access®, Beckman Coulter®) and an analysis of free fatty acids (FFA) was performed using spectrophotometry.

### Statistical analysis

The Shapiro–Wilk test was applied to determine whether the distribution curve was normal. The results are expressed as the mean ± standard deviation (*SD*) or standard error of mean (SEM). The data were compared using General Linear Model (GLM) with Newman Keuls *post-hoc* test or Student's *t-*test when necessary. The significance level was set at *P* ≤ 0.05. Statistical analyses were performed using Statistica 12.7 for Windows (StatSoft, Inc., Tulsa, USA). Specialist software was used for the calculation of the area under the curve (AUC) Origin® 8.5 (MicroCal Software, Inc., Westborough, MA, USA). The graphics were made using Prism® 6 software (GraphPad Software, Inc., La Jolla, CA, USA). Effect size was expressed as partial eta-squared (η^2^).

## Results

Table 1 presents the descriptive analysis data of the sample regarding age, height, body mass, and body mass index (BMI), the data obtained by the International Physical Activity Questionnaire (IPAQ), and the analyses of the food diaries filled in by volunteers during the training periods presented as average calories and macronutrients consumed per day. It is a sample composed of eutrophic young people, sufficiently active and with a balanced diet.

**Table 1:** Sample characteristics, IPAQ, and food diary.

Sample characteristics	Age (years)	23.67 ± 0.98
	Body Mass (kg)	73.61 ± 10.03
	Height (m)	1.74 ± 0.02
	BMI (kg/m^2^)	24.18 ± 3.35
IPAQ	Classification	Moderately active
Food diary	Calories/day (kcal)	2546.47 ± 756.47
	Carbohydrate (kcal)	1357.14 ± 604.13
	Protein (kcal)	500.89 ± 114.62
	Fat (kcal)	647.12 ± 156.38

*Data presented as mean ± SD, referring to 11 healthy males. BMI, Body Mass Index; IPAQ, International Physical Activity Questionnaire*.

Table 2 shows the data of the volunteers, obtained from the actigraph (average of variables taken over a period of 1 week), sleep questionnaire and polysomnography. The results show that the volunteers had good sleep quality, lasting more than 7 h per night. All sleep stages were preserved, respiratory indices remained within the normal range, and there were no reports of daytime sleepiness.

**Table 2:** Sleep characteristics.

Actigraphy	Awake time (minutes)	852.02 ± 97.08
	Sleep latency (minutes)	18.53 ± 8.76
	Sleep duration (minutes)	457.34 ± 52.87
	Sleep efficiency (%)	94.88 ± 1.59
Questionnaires	Pittsburgh sleep quality index	4.44 ± 3.12
	Sleep diary	23.88 ± 4.93
	Mini sleep questionnaire	25.33 ± 7.59
	Epworth sleepiness scale	7.55 ± 3.81
Polysomnography	Latency (minutes)	28.75 ± 32.23
	REM latency (minutes)	84.43 ± 35.34
	Total sleep time (minutes)	336.00 ± 77.60
	Sleep efficiency (%)	79.18 ± 16.60
	N1 (%)	8.85 ± 3.54
	N2 (%)	51.76 ± 6.45
	N3 (%)	21.65 ± 5.14
	REM (%)	18.73 ± 5.09
	Awake time (minutes)	58.98 ± 70.44
	Arousal	60.11 ± 23.14
	PLM (n°/h)	3.80 ± 7.72
	Respiratory events	18.44 ± 13.43
	RERA	12.22 ± 11.38
	RDI (n°/h)	3.64 ± 3.44
	AHI (n°/h)	1.00 ± 1.64

*Data presented as mean ± SD. REM, Rapid Eye Movements; N1, Stage 1; N2, Stage 2; N3, Stage 3; PLM, Periodic Limb Movements; RERA, Respiratory Effort Related Arousal; RDI, Respiratory Disturbance Index; AHI, Apnea Hypopnea Index*.

**Figure 2:**
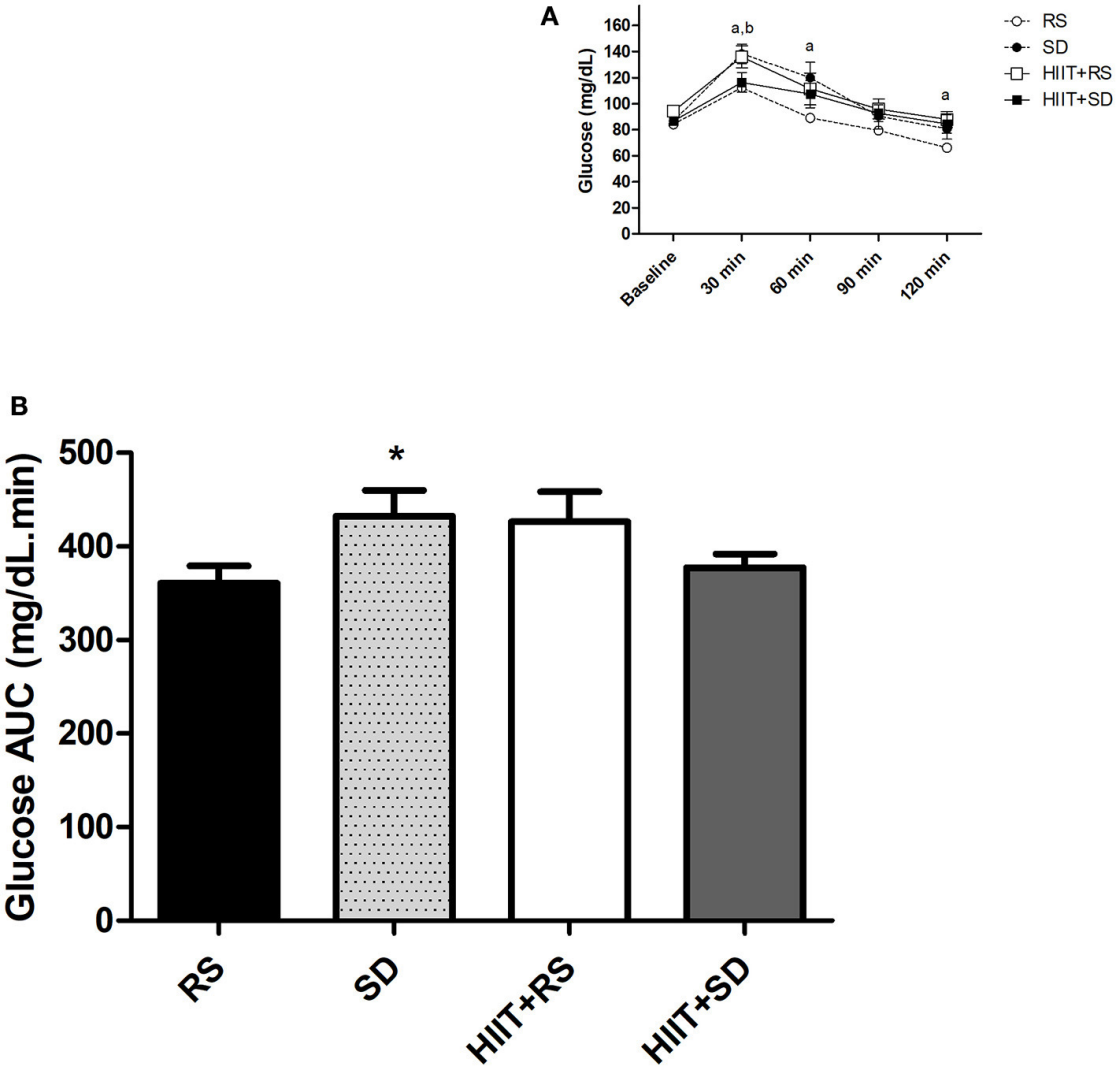
Glycemic curve and area under the curve of glucose. **(A)** Glycemic curve during OGTT and **(B)** AUC of Glucose after OGTT. GLM for repeated measures with *Post-Hoc* Newman Keuls Test. Data presented as mean ± SEM. ^*^Different to RS condition; ^a^RS condition different to *SD* condition; ^b^HIIT+*SD* condition different to *SD* condition. Min, minutes; AUC, Area under the Curve; OGTT, Oral Glucose Tolerance Test.

Figure 2A presents the results of the blood glucose. Comparing the groups at the baseline moment [*F*_(3.21)_ = 7.26, *P* < 0.01, η^2^ = 0.50], glycaemia levels in the HIIT+RS subjects were higher than in the other conditions (*p* < 0.01). After 30 min [*F*_(3.18)_ = 4.22; *P* < 0.01; η^2^ = 0.41] blood glucose in *SD* condition was higher than RS (*P* = 0.02) and HIIT+*SD* (*P* = 0.03). When tested at 60 min [*F*_(3.21)_ = 3.03, *P* = 0.05, η^2^ = 0.30] and 120 min [*F*_(3.21)_ = 2.56, *P* = 0.08, η^2^ = 0.26], blood glucose in *SD* condition was higher than the RS condition (*P* = 0.03).

In a group comparison (Figure 2A), the level of glycaemia in the RS condition [*F*_(4.40)_ = 17.53, *P* < 0.01, η^2^ = 0.67] was elevated for up to 30 min (*P* < 0.01), while blood glucose began to return to baseline values after 60 min (*P* < 0.01). In the *SD* condition [*F*_(4.40)_ = 15.09, *P* < 0.01, η^2^ = 0.62], blood glucose was higher after 30 min (*P* < 0.01) and 60 min (*P* < 0.01) when compared to baseline, while blood glucose began to return to baseline values after 90 min, continuing up to 120 min (*P* < 0.01). In the HIIT+RS condition [*F*_(4.40)_ = 11.74, *P* < 0.01, η^2^ = 0.59] and HIIT+*SD* condition [*F*_(4.40)_ = 6.56, *P* < 0.01, η^2^ = 0.45] the glycaemia levels were higher at 30 min than other moments (*P* < 0.01).

Figure 2B presents the results of AUC glucose during the OGTT. The glucose AUC [*F*_(3.21)_ = 3.52, *P* = 0.03, η^2^ = 0.69] was higher in the *SD* condition than in the RS condition (*P* = 0.03).

**Figure 3:**
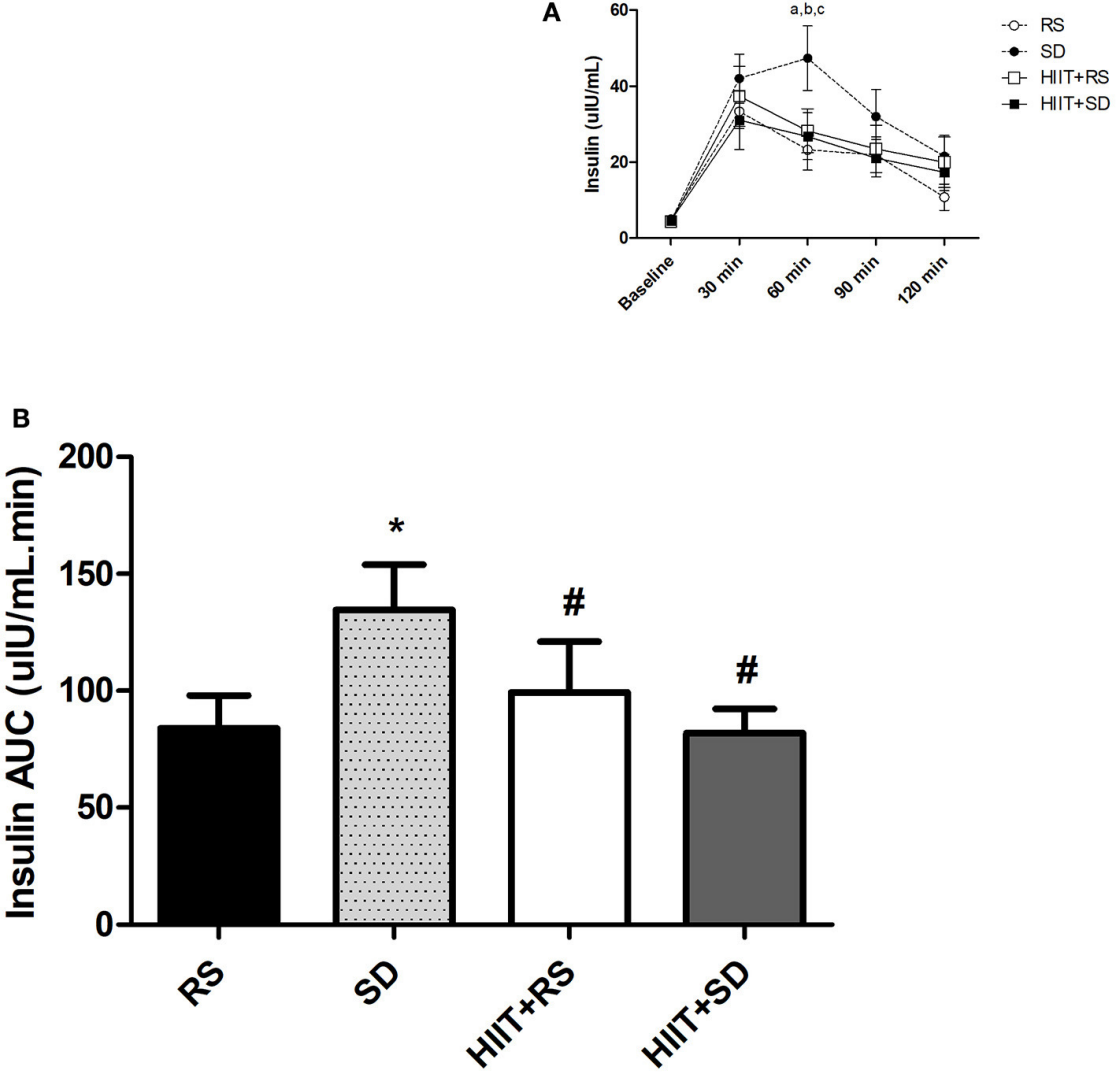
Insulin curve and area under the curve of insulin. **(A)** Insulin curve during OGTT and **(B)** AUC of Insulin after OGTT. GLM for repeated measures with *Post-Hoc* Newman Keuls Test. Data presented as mean ± SEM. ^*^Different to RS condition; ^#^Different to *SD* condition; ^a^*SD* condition different to RS condition; ^b^HIIT+*SD* condition different to *SD* condition; ^c^HIIT+RS condition different to *SD* condition. Min, minutes; AUC, Area Under the Curve; OGTT, Oral Glucose Tolerance Test.

Figure 3A presents the results of insulin concentrations. In comparison between groups, after 60 min [*F*_(3.15)_ = 5.70, *P* < 0.01, η^2^ = 0.87], insulin in *SD* condition was higher than other conditions (*P* = 0.01).

In group comparison (Figure 3A), the insulin in RS condition [*F*_(4.32)_ = 14.82, *P* < 0.01, η^2^ = 0.99] was elevated for up to 30 min (*P* < 0.01), was lower after 60 min (*P* = 0.02) and had returned to baseline values by 120 min (*P* = 0.01). In the *SD* condition [*F*_(4.36)_ = 12.10, *P* < 0.01, η^2^ = 0.99], insulin was higher after 30–90 min (*P* < 0.01) when compared to baseline moment, and began to return to baseline values after 120 min (*P* < 0.01). In the HIIT+RS condition [*F*_(4.28)_ = 9.76, *P* < 0.01, η^2^ = 0.99], insulin was elevated for up to 60 min (*P* < 0.01) and returned to baseline values after 90 min (*P* = 0.04). In the HIIT+*SD* condition [*F*_(4.32)_ = 5.61, *P* < 0.01, η^2^ = 0.95], insulin was elevated for up to 60 min (*P* < 0.01) and returned to baseline values after 90 min (*P* = 0.04).

Figure 3B presents the results of insulin AUC during the OGTT. The insulin AUC [*F*_(3.18)_ = 4.57; *P* = 0.01; η^2^ = 0.80] in *SD* was higher than other conditions (*P* = 0.02).

Calculations were performed: HOMA-IR Index [*F*_(3.35)_ = 0.66; *p* = 0.97; η^2^ = 0.84; RS = 1.03 ± 0.48; *SD* = 0.96 ± 0.47; HIIT+RS = 1.02 ± 0.43; HIIT+*SD* = 0.97 ± 0.40] and Matsuda Index (ISI) [*F*_(3.35)_ = 1.38; *p* = 0.26; η^2^ = 0.80; RS = 16.69 ± 8.23; *SD* = 10.98 ± 6.73; HIIT+RS = 11.92 ± 3.42; HIIT+*SD* = 13.35 ± 7.25]. However, there were no differences when the experimental conditions were compared.

**Figure 4:**
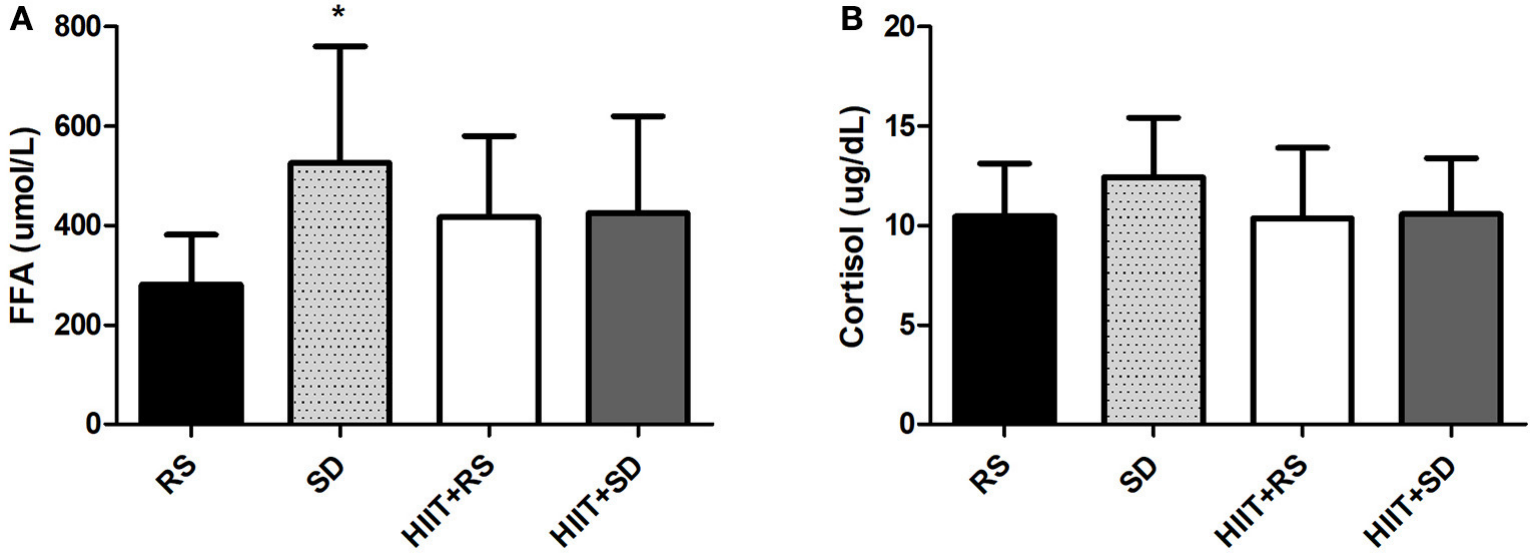
Free fatty acids and cortisol. **(A)** Free Fatty Acids concentrations and **(B)** Cortisol concentrations. GLM with *Post-Hoc* Newman Keuls Test. Data presented as mean ± *SD*. ^*^Different to RS condition.

Figure 4 shows the FFA and Cortisol concentrations. FFA [*F*_(3.21)_ = 3.34, *P* = 0.03, η^2^ = 0.67] had the highest concentration in the *SD* condition compared to RS condition (*P* = 0.02; Figure 4A), while Cortisol [*F*_(3.21)_ = 0.72, *P* = 0.54, η^2^ = 0.17] showed no significant differences between groups (Figure 4B).

**Figure 5:**
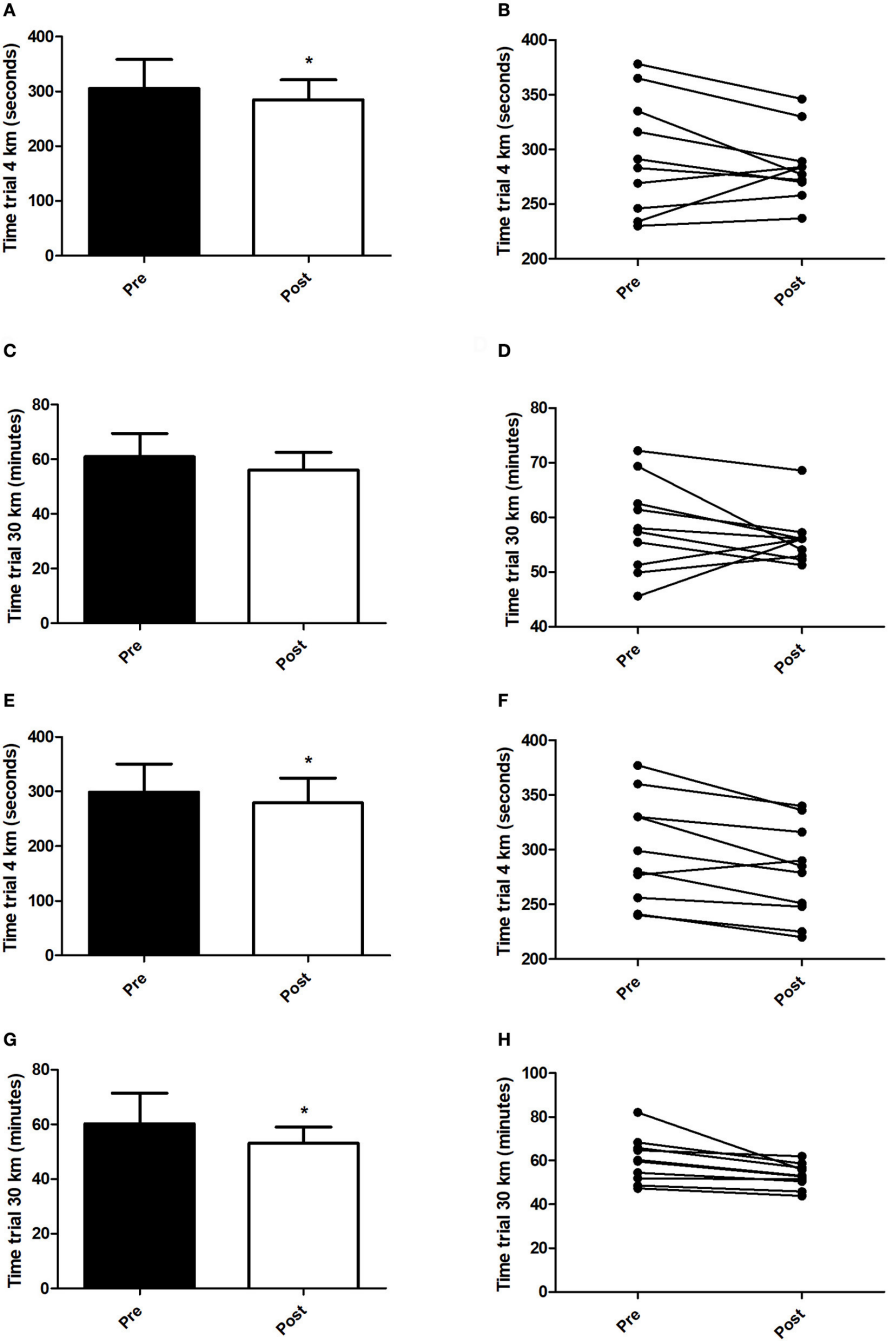
Time trials. **(A)** Mean of minutes in 4 km time trial test (HIIT+RS Condition), **(B)** Individual performance pre and post in 4 km time trial test (HIIT+RS Condition), **(C)** Mean of minutes in 30 km time trial test (HIIT+RS Condition), **(D)** Individual performance pre and post in 30 km time trial test (HIIT+RS Condition), **(E)** Mean of minutes in 4 km time trial test (HIIT+*SD* Condition), **(F)** Individual performance pre and post in 4 km time trial test (HIIT+*SD* Condition), **(G)** Mean of minutes in 30 km time trial test (HIIT+*SD* Condition), **(H)** Individual performance pre and post in 30 km time trial test (HIIT+*SD* Condition). Student *t*-Test. Data presented as mean ± *SD*. ^*^Different to pre training.

Figure 5 presents the performance data obtained in the tests against the time-trials tests. In HIIT+RS condition, the volunteers achieved faster times in 4 km time-trial test (Figure 5A; *t* = 2.53, *P* = 0.03), while there were no differences when compared pre- and post-training in 30 km time-trial test (Figure 5C). In HIIT+*SD* condition, the volunteers achieved faster times in both 4 km (Figure 5E; *t* = 3.43, *P* < 0.01) and 30 km (Figure 5G; *t* = 2.79, *P* = 0.02) time-trial tests.

## Discussion

The main findings of this study show that HIIT was effective in reversing the effects of sleep deprivation on glucose metabolism for 24 h, especially the improvement in levels of FFA, glycaemia and insulin during OGTT, suggesting that those who practice HIIT are not affected by the stress of sleep deprivation.

### Effects of sleep deprivation

The data confirm the negative impact of sleep deprivation on glucose metabolism. The decreasing availability of glucose for the tissues (Spiegel et al., 1999; González-Ortiz et al., 2000; Nedeltcheva et al., 2009; Donga et al., 2010), accompanied by reduced sensitivity of tissues to insulin (VanHelder et al., 1993; Tasali et al., 2008; Buxton et al., 2010; Klingenberg et al., 2013), is observed in different sleep deprivation protocols.

Changes in glucose metabolism following a period of sleep deprivation can be explained by decreased phosphorylation of PI3K/Akt that is closely linked to insulin signaling (Broussard et al., 2012). Literature shows that the increased activation of the HPA axis due to the stress of sleep debt, can influence the release of insulin to maintain normal glucose levels. Thus, increased cortisol is able to influence the response of glucose and insulin (Trenell et al., 2007) and sleep debt can increase serum concentrations of cortisol (Nedeltcheva et al., 2009; Buxton et al., 2010; Joo et al., 2012). This leads to activation of enzymes responsible for serine phosphorylation of both receptor substrates and proteins that participate in the transmission of the signal for translocation of the carrier to the membrane. This, in turn, blocks the transmission of the insulin signal and prevents the uptake of glucose into the cell (Leahy et al., 1986; Riboulet-Chavey et al., 2006). However, the results of this study appear to disprove the possibility that sleep debt can increase serum concentrations of cortisol, since this study found no differences in cortisol levels among the groups studied. Cortisol is a circadian hormone and its concentrations vary throughout the day (Michaud et al., 2009; Hayes et al., 2010). The lack of sequential samples of this hormone taken, and the time of collections (early morning), could explain the conflicting results.

Another possible explanation for this phenomenon is due to increased levels of FFA. The activation of the Central Nervous System can stimulate lipolysis, increasing the release of FFA into the bloodstream (Trenell et al., 2007). Literature shows that acute lack of sleep (Donga et al., 2010) or chronic sleep loss (Nedeltcheva et al., 2012) is able to increase the FFA levels. Furthermore, the increase of FFA is linked to serine kinase activation, which blocks the insulin signaling pathway, resulting in a lower translocation of glucose transporter to the cell membrane (Schenk et al., 2008). Our study found that in the *SD* condition there was an increase of 187.5% in FFA levels compared to the RS condition, reinforcing the fact that the insulin signaling pathway is impaired by sleep deprivation and, at least in part, due to high levels of FFA. This increase can mean a lower oxidation of fatty acids as an energy source during the agreed period, resulting in an accumulation of this substrate in the bloodstream. The mechanisms by which sleep debt increases the FFA concentrations are still uncertain. However, it is known that an increase of FFA implies a decrease in glucose uptake by tissues by a competitive mechanism (Randle, 1998), and the increase in fatty acids adversely impacts glucose transport (Dresner et al., 1999). After 4 consecutive nights of sleep restriction, Broussard et al. (2015) analyzed metabolic variables for 24 h, during which time there was an increase of 15–30% in FFA concentrations, especially in the period between 4 and 9 a.m., which coincides with the timing of collections of this study (between 7 and 8 a.m.).

### Effects of HIIT

Several studies show that in addition to improving performance, HIIT improves glucose metabolism, increases mitochondrial biogenesis, activity of respiratory chain and β-oxidation, and also the expression of GLUT4 and PGC-1α–all of which cause deterioration of insulin resistance (Burgomaster et al., 2005, 2008; Helgerud et al., 2007; Talanian et al., 2007; Daussin et al., 2008).

In healthy individuals, HIIT is able to increase insulin sensitivity (Richards et al., 2010), improve the action of insulin and lower the levels of FFA (Babraj et al., 2009). In individuals with insulin resistance risk, HIIT improves insulin sensitivity, increases the maximum oxygen intake, decreases body mass and body fat percentage (Earnest et al., 2013). In patients with diabetes mellitus type II, acute HIIT practice decreases glucose AUC (Gillen et al., 2012).

Considering glucose transporter in skeletal muscle, which is responsible for the glucose uptake into the muscle cell, several studies suggest the existence of different intracellular “pools” of GLUT4, which can be activated by insulin stimulation or exercise. Recent evidence indicates that these two mechanisms stimulate the translocation of the transporter to the cell membrane in a different way (Messina et al., 2015). During muscle contraction, proteins such as AMPK and Ras-related C3 botulinum toxin substrate 1 (Rac1) are activated (Rose and Richter, 2005). Once activated, these proteins initiate a cascade of reactions that lead to the translocation of GLUT4 to the cell membrane (Richter and Hargreaves, 2013; Sylow et al., 2013). It is noteworthy that both the activity of AMPK and Rac1 increases in line with the increase in exercise intensity (Chen et al., 2003; Gibala et al., 2009; Sylow et al., 2013), which may partly explain the success of HIIT for glycaemic control in individuals who have been deprived of sleep. Besides these factors, PGC1α can also participate directly in the regulation of GLUT4 expression in muscle cells (Michael et al., 2001; Baar et al., 2003). As reported in previous studies, HIIT induces increased AMPK and PGC1α activity, and translocation of GLUT4 (Burgomaster et al., 2005, 2008; Gibala et al., 2009; Little et al., 2010; Gillen et al., 2012; Sandvei et al., 2012). It is therefore suggested that the HIIT practiced by this study's volunteers was responsible for mitigating the deleterious changes arising from sleep deprivation.

In addition to the acute effect of exercise and activation of insulin-independent mechanisms to increase glucose uptake, regular physical exercise and its long-term benefits are also recorded in the insulin signaling pathway (Cartee et al., 1989). According to previous studies, exercise is able to activate Akt and improve glycaemic control by insulin receptor and insulin receptor substrate activation (Kirwan et al., 2000; Wojtaszewski et al., 2000; Luciano et al., 2002; Krisan et al., 2004), and it was considered that six sessions of HIIT were enough to cause such changes and thus improve glycaemic control in those subjects deprived of sleep.

Exercise, besides helping to improve glucose uptake, may help to decrease the action of factors that prevent glucose from entering the cell, as in the case of FFA. The increase in FFA, as for example, in mitochondrial dysfunction linked to insulin resistance (Kelley et al., 1999; Gaster et al., 2004), is associated with increased serine phosphorylation of protein kinases involved in the insulin pathway (Schenk et al., 2008). Moreover, exercise may increase mitochondrial biogenesis, resulting in increased performance as well as in the treatment of chronic diseases (Joseph and Hood, 2014), and more specifically, HIIT has been described as the only training mode capable of increasing mitochondrial biogenesis (Talanian et al., 2007; Little et al., 2010). In the *SD* condition it was noted that the FFA concentrations in the blood increased by 1.87 times, but when the volunteers had previously trained, the increase was lower at 1.51 times. Although these values are not statistically different, biologically these figures suggest that HIIT was able to increase mitochondrial biogenesis, thereby metabolizing energy substrates more efficiently and thus reducing the increase in both glucose and free fatty acids.

Finally, in addition to improving glucose uptake and FFA oxidation, it was observed that 2 weeks of HIIT was efficient in improving the performance of volunteers, corroborating other findings that also observed decreased times in time-trial tests (Gibala et al., 2006; Little et al., 2010). In the 4 km test, the volunteers decreased their times by 7% in the HIIT+RS condition and 9% in the HIIT+*SD* condition. In the 30 km test, the volunteers decreased their times by 1.2% in HIIT+RS condition and in the HIIT+RS condition by 11%. In addition to improved performance, other studies have shown an increase in VO_2_max (Whyte et al., 2010) and a decrease in body fat (Hazell et al., 2014), which confirms the effectiveness of HIIT in promoting benefits in a short period of time.

## Limitations

It is important to consider the potential role of some parameters that were not analyzed in this study, but which could interfere with the glucose metabolism responses to sleep deprivation, such as catecholamine concentrations (Trenell et al., 2007) and melatonin (Zanuto et al., 2013).

In addition, this study has some limitations, such as the population studied and the sample size which means that it is not feasible to extrapolate the results to other populations, the non-randomization of experimental conditions, and the method by which insulin resistance (OGTT) and the non-standardization of volunteers' nutritional intake were evaluated.

## Conclusion

It was concluded that HIIT prior to sleep deprivation was able to attenuate the increase in glucose, insulin and FFA in the blood. Therefore, this method produces significant metabolic changes and could be considered as a non-pharmacological strategy which is able to minimize insulin resistance imposed by sleep deprivation.

## Author contributions

JdS, MD, MdM, ST, and HA: Substantial contributions to the conception or design of the work; JdS, MD, and HA: Acquisition, analysis, or interpretation of data for the work; JdS MD, HA: Drafting the work; JdS, MD, MdM, ST, HA: Revising it critically for important intellectual content; JdS, MD, MdM, ST, HA: Final approval of the version to be published; JdS, MD, MdM, ST, and HA: Agreement to be accountable for all aspects of the work in ensuring that questions related to the accuracy or integrity of any part of the work are appropriately investigated and resolved.

### Conflict of interest statement

The authors declare that the research was conducted in the absence of any commercial or financial relationships that could be construed as a potential conflict of interest.
